# Multiple aetiologies of the progressive dysexecutive syndrome and the importance of biomarkers

**DOI:** 10.1093/braincomms/fcaa127

**Published:** 2020-11-01

**Authors:** David T Jones

**Affiliations:** 1 Department of Neurology, Mayo Clinic, Rochester, MN 55902, USA; 2 Department of Diagnostic Radiology, Mayo Clinic, Rochester, MN 55902, USA

On behalf of my co-authors, I would like to thank Tábuas-Pereira *et al.* (2020) for their letter related to our recent article (Townley *et al.*, 2020). In this letter, Tábuas-Pereira *et al.* describe a cohort of patients with *GRN* mutations that have similarities with the progressive dysexecutive syndrome we recently described in individuals with Alzheimer’s disease. When using the A/T/(N) staging scheme (Jack *et al.*, 2018), none of the *GRN* cases reported by Tábuas-Pereira *et al.* were A+/T+/(N)+ or A+/T+/(N)− in contrast to our cases where 48/55 (87%) had A+/T+/(N)+ profiles. If we had tau-PET in the seven cases that were A+/T−/(N)+, rather than only cerebrospinal fluid (CSF) p-tau, we suspect that they would have been found to be T+ as this was the finding for all similar cases with tau-PET available that were T- by CSF (*n* = 5). Therefore, we concur with Tábuas-Pereira *et al.* that the aetiology of the progressive dysexecutive syndrome described in their cohort is physiologically distinct from sporadic Alzheimer’s disease despite similar clinical syndromes in the two cohorts. This highlights the importance of defining aetiologically agnostic clinical syndromes with the subsequent use of aetiologically specific biomarkers. In fact, some of us have reported similar circumstances when looking at individuals presenting with the logopenic variant of primary progressive aphasia who were amyloid-PET negative (Josephs *et al.*, 2014). Half of these cases were found to be carriers of *GRN* mutations. We have made conceptually similar insights about TDP-43-associated neurodegeneration utilizing biomarkers to better understand the aetiology of the amnestic dementia clinical syndrome. In such clinical cases, the absence of positive tau biomarkers with distinctive neurodegeneration profiles on ^18^F-fluorodeoxyglucose-PET, irrespective of amyloid status, was found to have limbic-predominant age-related TDP-43 encephalopathy (Botha *et al.*, 2018*a*, *b*; Nelson *et al.*, 2019). Therefore, the absence of tau biomarkers, even in the presence of amyloid biomarkers, should be an indicator of a potential TDP-43-related aetiology in some clinical syndromes more commonly caused by Alzheimer’s disease (i.e. amnestic dementia, logopenic variant of primary progressive aphasia and progressive dysexecutive syndrome). However, a subset (*n* = 5) of our cases were tau negative in the CSF and tau positive by PET and this discrepancy should be considered when interpreting biomarkers within the proper clinical context.

We similarly have observed patients presenting with a progressive dysexecutive syndrome from a wide variety of aetiologies, including *GRN* mutation carriers as described by Tábuas-Pereira *et al.* In our experience, we have found that the parietofrontal hypometabolism observed in these *GRN* cases is strikingly asymmetric in comparison to the hypometabolism observed in sporadic Alzheimer’s disease cases with the same clinical syndrome. Although asymmetry is common in the Alzheimer’s disease cases we reported, the less involved contralateral hemisphere was rarely spared to the degree that it is in *GRN* mutation carriers. Such parietofrontal asymmetry in neurodegeneration in the setting of positive family history and negative biomarkers for Alzheimer’s disease should raise the strong possibility of a *GRN* mutation. We have also seen this profile in individuals with *DNMT1* mutations (Bi *et al.*, 2020). We agree with Tábuas-Pereira *et al.* that not all biomarker profiles are indicative of a sporadic Alzheimer’s disease aetiology in individuals with a progressive dysexecutive syndrome, but we do believe some are. In particular, observing a A+/T+/(N)+ profile in individuals presenting with a progressive dysexecutive syndrome without a strong family history and lacking striking asymmetry makes the presence of a *GRN* mutation unlikely. More research on this topic is certainly required.

We would suggest caution before assigning a frontoparietal anatomic moniker to the progressive dysexecutive syndrome. This is because the core executive functions (working memory, cognitive flexibility, and inhibition) likely have variable anatomic correlates within and across individuals. While a working memory predominant progressive dysexecutive syndrome may have a tight association with parietofrontal functional anatomy as suggested by our prior work (Jones *et al.*, 2017), more research is required to establish the fidelity of this hypothesized relationship.
